# Supplementary material for: Content-rich biological network constructed by mining PubMed abstracts
Source: BMC Bioinformatics. 2004 Oct 8;5:147. doi: 10.1186/1471-2105-5-147 (PMC528731; doi:10.1186/1471-2105-5-147)
Supplement: Additional File 2 — The original results of the above study (non-essential files are deleted to keep the file size under the limit set by BMC bioinformatics). [file 1471-2105-5-147-S2.bz2 › chilibotAdditionalFile2/dip05/238ID9160889E779/html/USF1_FOSL1.html]

 


 **USF1** and **FOSL1** 
  
Found 2 abstracts in PubMed, retrieved 2.  
 

 What does Google say? 
 PDF only 
| .edu only 

---

**Interactive relationship** (e.g. stimulation, inhibition, etc)

**Neutral relationship**- Cross family interaction between the bHLHZip USF  [ **USF1** ]  and bZip Fra1  [ **FOSL1** ]  proteins results in down regulation of AP1 activity.  Ref: 9160889 Oncogene, 1997
- Both the bHLHZip and the N terminal regions of Fra1  [ **FOSL1** ]  are required for efficient interaction with USF  [ **USF1** ] .  Ref: 9160889 Oncogene, 1997
- USF  [ **USF1** ]  specifically interacts with Fra1  [ **FOSL1** ]  but not with other closely related family members, c Fos, Fra2, FosB, or with c Jun.  Ref: 9160889 Oncogene, 1997
- A bZip protein, Fra1  [ **FOSL1** ] , was found to efficiently interact with USF  [ **USF1** ] .  Ref: 9160889 Oncogene, 1997

**Non-interactive relationship** (e.g. studied together, co-existance, homology, etc.)

- In vivo association between USF  [ **USF1** ]  and Fra1  [ **FOSL1** ]  has been demonstrated by co immunoprecipitation.  Ref: 9160889 Oncogene, 1997
- These data show that USF  [ **USF1** ]  and Fra1  [ **FOSL1** ]  physically and functionally interact demonstrating that cross talk occurs between factors of distantly related transcription families.  Ref: 9160889 Oncogene, 1997
